# Arterialized Venous Bone Flaps: An Experimental Investigation

**DOI:** 10.1038/srep31970

**Published:** 2016-08-25

**Authors:** Farzad Borumandi, James P. Higgins, Heinz Buerger, Anna Vasilyeva, Memmet Emre Benlidayi, Leman Sencar, Alexander Gaggl

**Affiliations:** 1 Department of Oral and Maxillofacial Surgery, Paracelsus Medical University, Muellner Hauptstrasse 48, A-5020 Salzburg, Austria; 2 Department of Oral and Maxillofacial Surgery, Queen Victoria Hospital, Holtye Road, East Grinstead, RH19 3DZ, United Kingdom; 3 The Curtis National Hand Center, MedStar Union Memorial Hospital, 3333 North Calvert Street, Johnston Professional Building, Baltimore, MD 21218, USA; 4 University Hospital Graz, Auenbruggerplatz 29, 8036 Graz, Austria; 5 Department of Oral and Maxillofacial Surgery, Dental Faculty, Çukurova University, Adana, Turkey; 6 Department of Histology, Faculty of Medicine, Çukurova University, Adana, Turkey

## Abstract

In arterialized venous flaps (AVFs) the venous network is used to revascularize the flap. While the feasibility of AVFs in soft tissues has been reported there is no study on osseous AVFs. In this study we aim to assess the flap survival of osseous AVFs in a pig model. Medial femoral condyle flaps were elevated in 18 pigs. Three groups were created: AVF (n = 6), conventional arterial flap (cAF, n = 6) and bone graft (BG, n = 6). The AVFs were created by anastomosis of genicular artery with one vena comitans while leaving one efferent vein for drainage. After 6 months the specimens were harvested. The histology and histomorphometry of of the bone in cAF and AVF was significantly superior to bone grafts with a higher bone volume in AVFs (p = 0.01). This study demonstrates that osseous free flaps may be supported and survive using the technique of arterialization of the venous network. The concept of AVFs in osseous flaps may be feasible for revascularization of free flaps with an inadequate artery but well developed veins. Further experimental and clinical studies are needed to assess the feasibility of clinical use of arterialized venous bone flaps.

Arterialized venous flaps (AVFs) are tissue flaps harvested without conventional vascular pedicles. Perfusion of the flap is achieved by anastomosis of a recipient artery to the venous plexus within the flap. AVFs were firstly introduced in an experimental study on rats by Nakayama *et al.* in 1981^1^. Subsequent authors have reported on various techniques of arterialization of the venous system. Variations have been reported in the arterial inflow technique (configuration of inflow vein(s), direction of inflow valves) as well as the means of providing outflow (efferent vein(s) or flowthrough artery)^2^,^3^. Experimental and clinical studies have demonstrated the feasibility and relative reliability of various AVF techniques^4^,^5^,^6^,^7^,^8^,^9^,^10^,^11^.

There is no consensus on the mechanisms of tissue perfusion in AVFs. Authors have suggested that flap survival may be explained via arterio-venous shunting^11^, reversal of flow from the venules into the capillary system^12^, or oxygenation through the venous system without entering the arterial system^13^.

Although the mechanism of successful tissue perfusion of AVFs is unclear, clinical and experimental experience has confirmed skin flap survival using this unconventional technique. This has widened the scope of possible donor sites for skin flap harvest and minimized donor site morbidity.

It is unknown whether the potential advantages of this technique could be employed in flaps containing other tissue types. The goal of this study is to similarly assess the feasibility of AVFs for the osseous flap perfusion via arterialization of the venous system.

## Material and Methods

The animal experiments were conducted according to the guidelines of animal welfare of the World Organization for Animal Health, and approved by the local animal ethics committee of Cukurova University in Adana, Turkey. Eighteen adult, 6- to 8-month-old domestic pigs bred at the Medical Experimental Research Center (DETAUM) at Çukurova University were used in this study. At surgery, their body weight was 40 ± 5 kg. They were kept on a high-calorie feed and allowed water ad libitum. The osseous segments were raised from the medial femoral condyle. Three different bone segments were created: conventional arterial bone flaps (cAF, n = 6), the arterialized venous bone flaps (AVF, n = 6) and non pedicled bone grafts as a control group (BG, n = 6). Each pig had one of the three bone segments created using the left femur. At conclusion of the procedure the pigs were permitted to return to ambulation and maintained for 6 months.

All the pigs were sacrificed after 6 months of healing by an intravenous lethal dose of thiopental sodium and potassium.The harvested specimens were decalcified and prepared for histomorphometric and histologic assessment.

### Surgical Method

The pigs were pre medicated with intramuscular injections of 20 mg/kg ketamine (Alfamyne, Egevet, İzmir, Turkey), 2 mg/kg xylazine (Rompun, Bayer, İstanbul, Turkey), and 0.5 mg atropine sulphate. General anesthesia was performed with intravenous administration of 15 mg/kg Thiopental Sodium (Pental Sodyum, I.E. Ulagay, İstanbul, Turkey). As a prophylactic antibiotic, 1 g Cephazolin Sodium (Sefazol, Mustafa Nevzat İlaç, İstanbul, Turkey) was administered preoperatively to all the pigs intravenously.

**Figure 1:**
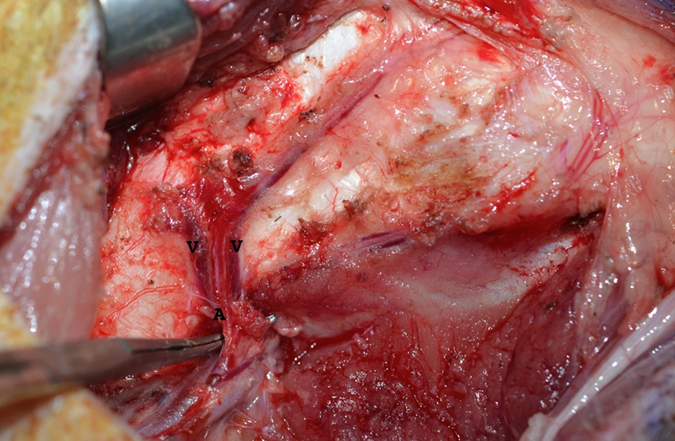
Demonstrates the medial femoral condyle left femur. The Pedicle is dissected and the descending genicular artery and accompanying venae comitans are demonstrated.

The distal femur was used to raise the medial femoral condyle flap based on the descending genicular artery and its venae comitans as previously described^14^,^15^. An 8–10 cm longitudinal incision was performed in the medial aspect of the leg. The vastus medialis muscle was reflected anteriorly exposing the medial femur. The periosteal vessels and the descending genicular artery and accompanying veins were dissected (Fig. 1).

**Figure 2:**
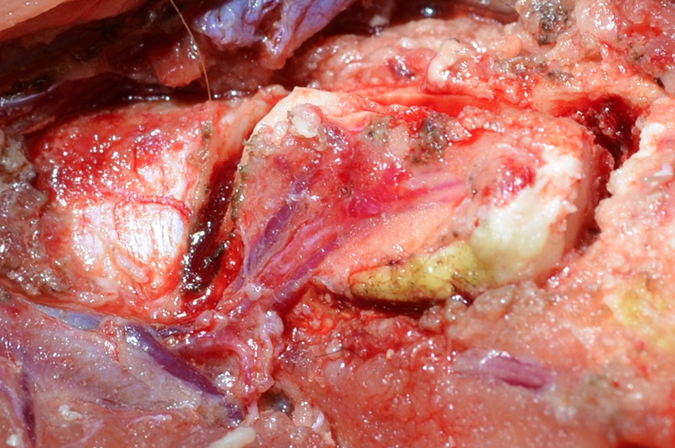
Demonstrates the elevated medial femoral condyle osseous flap. The distal end of the pedicle is ligated.

**Figure 3:**
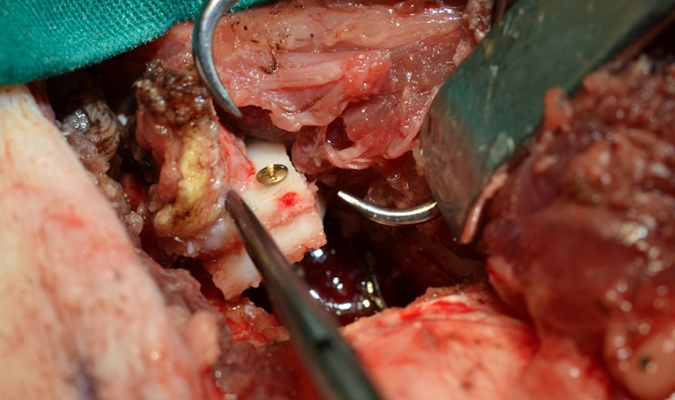
Demonstrates the vascularized medial condyle flap which has been secured with a non-vascularized bone block creating a bone chamber.

**Figure 4:**
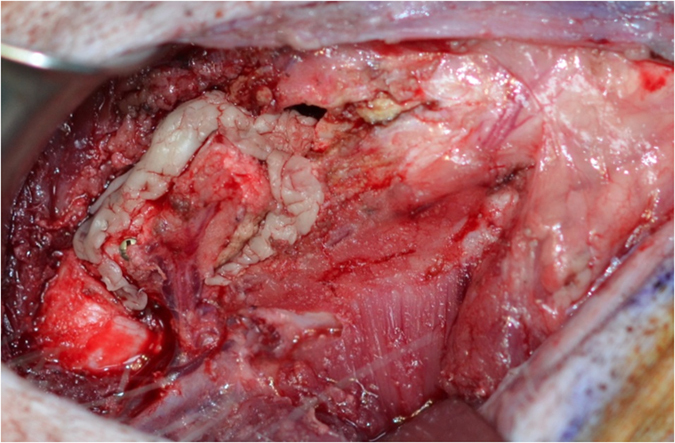
The flap was isolated from revascularization from surrounding bone by lining the donor site with a layer of bone cement and the flap was returned to its original location.

In the group with conventional arterial flap (cAF) the pedicle was left intact and the continuity of the periosteum was preserved. The distal end of the artery and venae comitans were ligated and the corticoperiosteal flap was elevated using a Fisher burr. (Figure 2) From the adjacent femur shaft and in the same size of the bone flap, a monocortical bone block was harvested. The harvested bone graft was placed beneath the bone flap with the endosteal site facing up. The bone flap/ bone graft sandwich was secured with a 12 mm lag screw (Synthes) creating a chamber, where eventually newly generated bone can grow into (Fig. 3). A thin layer of bone cement was used to line the defect created in the femur shaft and the bone flap was embedded into the cement. The cement layer was applied in order to prevent vascular ingrowth to the experimental bone segments from the underlying adjacent medullary canal of the femur (Fig. 4).

**Figure 5:**
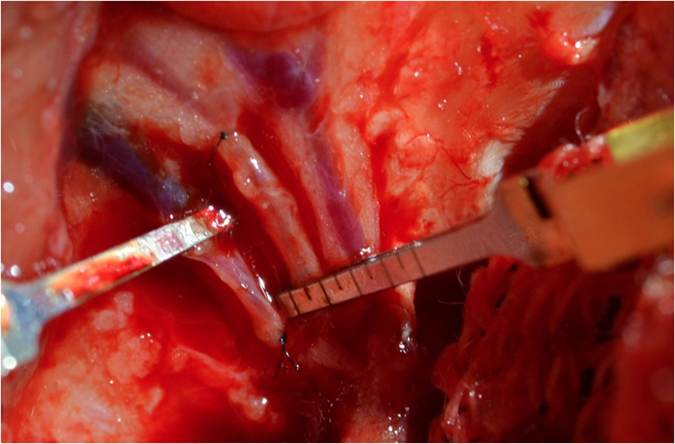
Demonstrates the preparation of the vessels prior to arterio-venous anastomosis. The descending genicular artery is ligated distally and clamped proximally. One vena comitans is clamped distally and ligated proximally. The second vena comitans is left as efferent vein for drainage.

**Figure 6:**
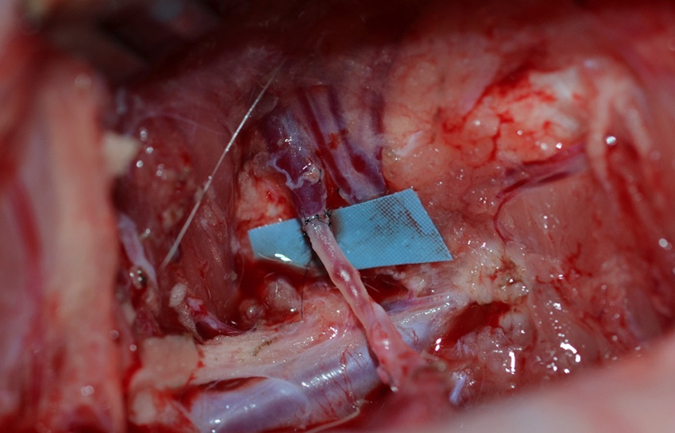
The Arterio-Venous Anastomosis is demonstrated.

In the AVF group the arterial pedicle and one of the two venae comitans were purposefully transected. The second vena comitans remained untouched (Fig. 5). The genicular artery was anastomosed with the transected vena comitans using 11.0 Ethilon and surgical loupes (Fig. 6). The second vena comitans that was not transected served as the draining vein. All anastomosis were performed by co-authors, who are experienced microvascular surgeons (AG, HB, JH). After successful anastomosis the periosteum was incised, the distal end of the pedicle was ligated and the bone flap was raised in the same fashion as described above. A bone chamber was created which was returned to the harvest site after a thin layer of bone cement was used to line the defect similar to the conventional flap group described above.

In the control group a bone chamber was created with two non-vascularized bone grafts and it was insulated in the same fashion as described above.

In all three groups the knee joint remained closed throughout the dissection. After the procedures were complete a layered wound closure was performed.

### Histological Methods

The specimens obtained from each group were fixed in 10% formalin and decalcified in 5% nitric acid for histologic examination. After being embedded in paraffin, 5-μm-thick sections were prepared and stained with hematoxylin and eosin. Two slides from each specimen were examined by 2 histologists blinded to the groupings.

Development of lamellar bone and bone trabeculae, the presence of osteocytes in bone lacunae, regions with cartilage-bone transformation, presence of blood vessels in Haversian systems were analysed for assessment of bone viability.

The presence of osteocytes in lacunae, appearance of the surrounding cellular activity, and the presence of interstitial lamellar osteocytes were evaluated in the sections. Histologic evaluation of necrosis and viability was done according to these criteria.

### Histomorphometric Analysis

The digital images of histological slides were obtained with a digital camera (Olympus DP 70, Tokyo, Japan) attached to a microscope (Olympus BX50, Tokyo, Japan) at a magnification of ×4. The obtained images were transferred to a personal computer and WinTAS (Trabecular Analyze System, version 1.2.9) was used for histomorphometric analysis. The following parameters were assessed: the percentage of bone volume to the total measured volume (BV/TV), trabecular thickness (TbTh), trabecular number (TbN), and trabecular separation (TbSp). These acronyms and abbreviations were proposed by the American Society for Bone and Mineral Research Committee.

### Statistical Analysis

All analyses were performed using SPSS 20.0 statistical software package (IBM SPSS Statistics). Continuous variables were summarized as mean and standard deviation and as median and minimum-maximum where appropriate. For comparison of continuous variables between two groups, Mann Whitney U test was used. The Kruskal Wallis test was used to compare more than two groups. The statistical level of significance for all tests was considered to be 0.05. The given p values were not adjusted for multiple comparison.

## Results

### Histological findings

Specimens of all bone groups were histologically examined for quality and viability of the bone. Unfortunately two of the animals died during the postoperative course. Since we had performed arterial flaps and bone grafts on those animals the number of specimens in cAF and BG group was reduced.

There was no significant difference to observe histologically between the cAF (n = 4) and AVF (n = 6) groups. The bone in both groups was covered by periosteum that consisted an outer fibrous layer with irregular dense connective tissue and an inner more cellular layer. The underlying bone was largely composed of Haversian systems with blood vessels and nerve fibers.

**Figure 7:**
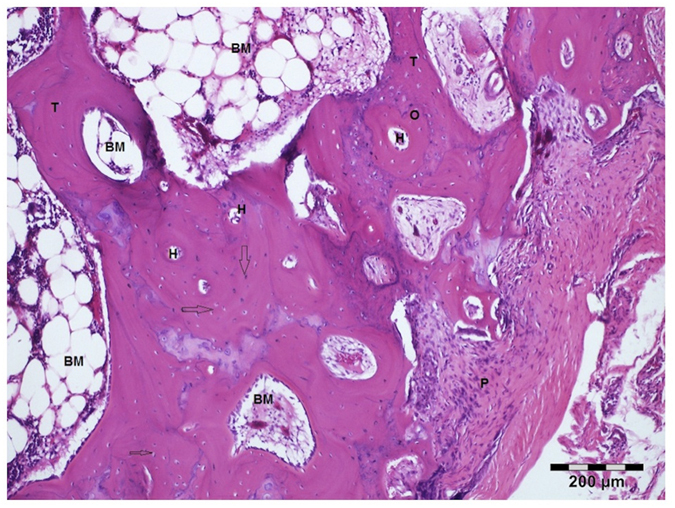
Decalcified section of the AVF group. P, periosteum; O, osteon; H, Haversian canal; T, trabeculae; arrows, osteocyte in lacunae; BM, bone marrow. Hematoxylin and Eosin stain. Bar = 200 μm.

Some cartilaginous islets were seen within the bone trabeculae in AVFs with a loose network of bone marrow that included hemopoietic cells and adipose cells (Fig. 7). In both groups the lamellar system was well developed with evidence of viable osteocytes in the lacunae and blood vessels in Haversian canals.

**Figure 8:**
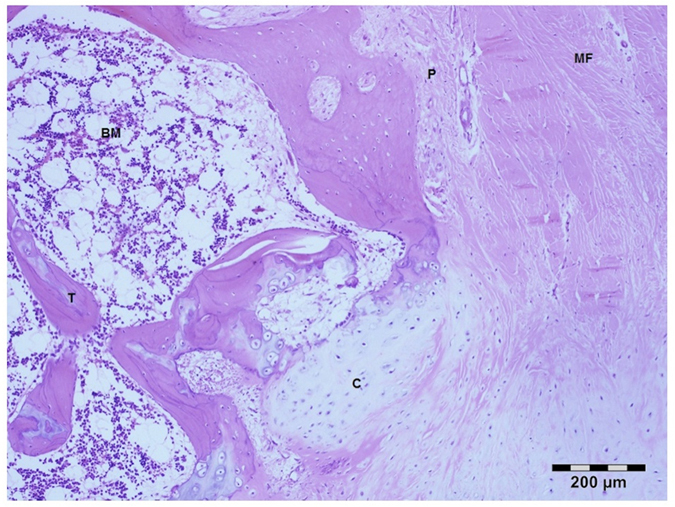
Decalcified section of the BG group. P, periosteum; MF, muscle fiber; C, cartilage tissue; T, trabeculae; BM, bone marrow. Hematoxylin and Eosin stain. Bar = 200 μm.

In the bone graft group (n = 4) the bone thickness was noticeably reduced. Unlike the vascularized bone flaps the bone lamellar system was not well developed. There was a reduced number of Haversian systems with bone canals in which blood vessels and nerve fibers were present. The bone trabeculae were partially fused with multiple cartilaginous islets. There were quite large bone cavities that contained hemopoietic tissues and loose connective tissue between trabeculae. Furthermore in the BG group, the lamellar system and the blood vessels in the Haversian canals were not well developed with a reduced number of viable osteocytes in the lacunae (Fig. 8).

### Histomorphometric Analysis

**Figure 9:**
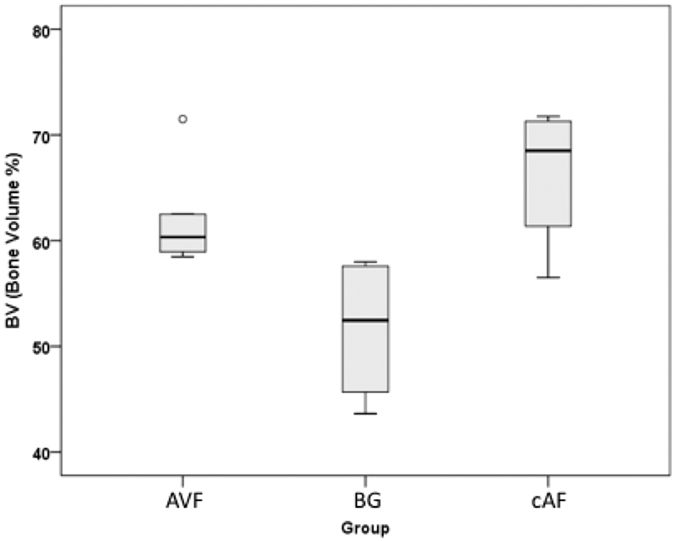
Histomorphometric parameters assessed in all groups. The percentage of bone volume in all 3 groups is demonstrated.

In histomorphometric assessment of bone architecture the percentage of bone volume to total measured volume (BV/TV) was decreased in bone grafts compared with AVFs (P = 0.01) and cAFs (p = 0.11) Fig. 9. The bone trabeculae were significantly thinner in the BG group (mean 18.38 μm) compared to the cAF group (mean 26.93 μm, P = 0.029). In AVF group the trabecular thickness was greater than the BG group with a low significance level (mean 24.18 μm, p = 0.17). No significant difference in bone volume or trabecular thickness was found between the two vascularized groups cAF and AVF (Table 1).

The trabecular separation was greater in BG group (mean 18.13 μm) which was significantly higher than in AVF group (13.52 μm, p = 0.038) and statistically not significant in cAF group (13.32 μm, p = 0.20).

The trabecular number was higher in the vascularized AVF and cAF groups compared to the non vascularized BG group nevertheless with a weak statistical correlation (p = 0.17, p = 0.34).

**Figure 10:**
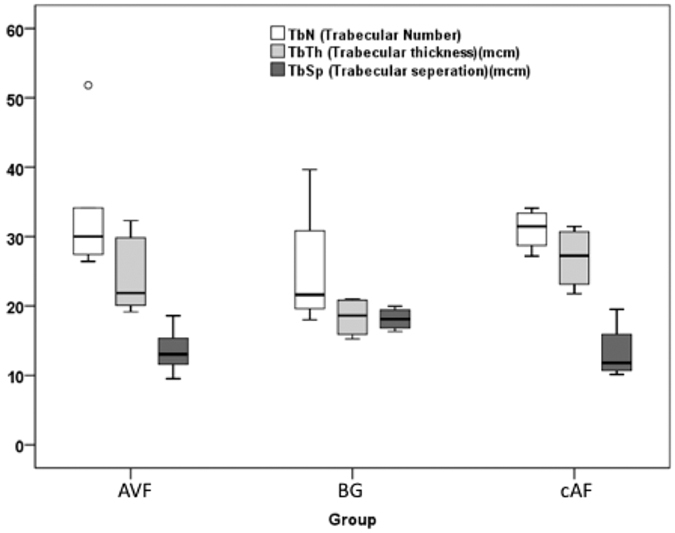
Histomorphometric parameters assessed in all groups.

There was no significant difference in trabecular number or in trabecular seperation between the two vascularized groups AVF and cAF (Fig. 10).

## Discussion

In arterialized venous flaps the venous network is used to perfuse the harvested tissues. Although the exact mechanism of successful tissue perfusion in AVFs is unclear, various clinical and experimental studies have confirmed skin flap survival using this unconventional technique^4^,^5^,^7^,^10^,^16^,^17^,^18^,^19^. AVFs may be harvested in a wide range of locations. The harvest is technically less demanding and more superficial than conventional arterial pedicle flaps, permitting rapid harvest of very thin tissue^5^,^7^,^8^,^10^. The advantages gained by this technique in transfer of skin flaps brings into question whether other tissues could be perfused via arterialization of the venous system. AVFs have been successfully used as composite soft tissue flaps in soft tissues encompassing fascia and tendons^20^. In this study we aimed to assess the flap survival and bone quality of osseous AVFs.

In this pig model arterialized venous medial femoral condyle flaps (AVFs), were created by anastomosing the arterial pedicle to one of the venae commitans while leaving one efferent vein for drainage. The flap was isolated from revascularization from surrounding bone by lining the donor site with a layer of bone cement prior to returning the arterialized venous bone flap to its original location. Similar steps were taken to create cAFs and BGs, and return them to their original donor site with cement lining. Microscopic evaluation of each group after 6 months revealed no significant difference in healing and bone quality in the vascularized groups (AVF and cAF). The histology and histomorphometry of vascularized bone flaps was significantly superior to bone grafts with a higher bone volume in AVFs (p = 0.01). This study suggests that osseous free flaps may be supported and survive using the technique of arterialization of the venous network. The concept of AVFs in osseous flaps may be useful for revascularization of free flaps with an inadequate artery but well developed veins.

This study was limited by the small number of animals included in each group and the small size of osseous flaps created. Several questions warrant further experimental or clinical studies. Prior to clinical application of this technique it would be helpful to determine if the dimensions of the osseous flap would alter survival of arterialized venous bone flaps. Furthermore, while this study demonstrated survival and promising histomorphometric characteristics of these arterialized bone flaps, our model did not determine whether this type of perfusion would impact capability and speed of bone healing if applied in cases of fracture or nonunion repair.

## Conclusion

This experimental study of arterialized venous bone flaps demonstrated flap survival with equally well developed bone quality compared with conventional arterial bone flaps. The bone volume of osseous AVFs was significantly higher than in bone grafts. Further experimental and clinical studies are needed to assess the feasibility of clinical use of arterialized venous bone flaps.

## Additional Information

**How to cite this article**: Borumandi, F. *et al.* Arterialized Venous Bone Flaps: An Experimental Investigation. *Sci. Rep.* **6**, 31970; doi: 10.1038/srep31970 (2016).

## Figures and Tables

**Table 1:** Histomorphometric results of all groups.

Variable	AVF Group (n = 6)	BG Group (n = 4)	cAF Group (n = 4)	p-value 1,2	p-value 1,3	p-value 2,3
BV/TV %						
Mean ± SD	62.01 ± 4.86	51.63 ± 7.09	66.33 ± 6.98	0.010*	0.476	0.114
Median	60.34	52.45	68.52			
Min - Max	58.47–71.49	43.62–57.99	56.50–71.76			
TbTh (μm)						
Mean ± SD	24.18 ± 5.51	18.38 ± 2.89	26.93 ± 4.55	0.171	0.352	0.029*
Median	21.86	18.62	27.23			
Min - Max	19.15–32.27	15.26–21.01	21.77–31.46			
TbN (mm^−1^)						
Mean ± SD	33.30 ± 9.48	25.23 ± 9.76	31.05 ± 3.01	0.171	0.999	0.343
Median	30.02	21.62	31.46			
Min - Max	26.42–51.81	18.04–39.64	27.19–34.09			
TbSp (μm)						
Mean ± SD	13.52 ± 3.17	18.13 ± 1.61	13.32 ± 4.22	0.038*	0.762	0.200
Median	13.05	18.11	11.82			
Min - Max	9.52–18.59	16.33–19.96	10.14–19.50			

(cAF, conventional arterial flap; AVF, arterialized venous flaps; BG, bone graft; BV/TV, percantege of bone volume to total measured volume; TbTh, trabecular thickness; TbN, trabecular number; TbSp, trabecular separation).

^*^Statistically significant at p < 0.05.

## References

[b1] Nakayama Y., Soeda S. & Kasai Y. Flaps nourished by arterial inflow through the venous system: an experimental investigation. Plast Reconstr Surg. 67, 328–34 (1981). 7232566 10.1097/00006534-198103000-00009

[b2] Woo S. H. *et al.* A retrospective analysis of 154 arterialized venous flaps for hand reconstruction: an 11-year experience. Plast Reconstr Surg. 119, 1823–38 (2007). 17440363 10.1097/01.prs.0000259094.68803.3d

[b3] Goldschlager R. *et al.* The nomenclature of venous flow-through flaps: updated classification and review of the literature. Microsurgery. 32, 497–501 (2012). 22434451 10.1002/micr.21965

[b4] Garlick J. W. *et al.* Arterialized venous flow-through flaps in the reconstruction of digital defects: case series and review of the literature. Hand (N Y). 10, 184–90 (2015). 26034428 10.1007/s11552-014-9684-0 PMC4447646

[b5] Kayalar M. *et al.* Clinical applications of free arterialized venous flaps. J Plast Reconstr Aesthet Surg. 67, 1548–56 (2015). 24961162 10.1016/j.bjps.2014.05.061

[b6] Yan H. *et al.* The effect of hemodynamic remodeling on the survival of arterialized venous flaps. PLoS One. 8, e79608 (2013). 24265782 10.1371/journal.pone.0079608 PMC3827173

[b7] Iglesias M. *et al.* Arterialized venous free flap for reconstruction of burned face. Microsurgery. 28, 546–50 (2013). 18683867 10.1002/micr.20525

[b8] Kovacs A. F. Comparison of two types of arterialized venous forearm flaps for oral reconstruction and proposal of a reliable procedure. J Craniomaxillofac Surg. 26, 249–54 (1998). 9777504 10.1016/s1010-5182(98)80021-8

[b9] Woo S. H. *et al.* Effects of blood flow and venous network on the survival of the arterialized venous flap. Plast Reconstr Surg. 101, 1280–9 (1998). 9529214 10.1097/00006534-199804050-00019

[b10] Yilmaz M. *et al.* Free arterialized venous forearm flap. Ann Plast Surg. 34, 88–91 (1995). 7702310 10.1097/00000637-199501000-00019

[b11] Chavoin J. P. *et al.* Island flaps with an exclusively venous pedicle. A report of eleven cases and a preliminary haemodynamic study. Br J Plast Surg. 40, 149–54 (1987). 3567447 10.1016/0007-1226(87)90187-1

[b12] Baek S. M. *et al.* Experimental studies in the survival of venous island flaps without arterial inflow. Plast Reconstr Surg. 75, 88–95 (1985). 3966113 10.1097/00006534-198501000-00020

[b13] Matsushita K. *et al.* Blood flow and tissue survival in the rabbit venous flap. Plast Reconstr Surg. 91, 127–35 (1993). 8416517

[b14] Gaggl A., Burger H. & Chiari F. M. The microvascular osteocutaneous femur transplant for covering combined alveolar ridge and floor of the mouth defects: preliminary report. J Reconstr Microsurg. 24, 169–75 (2008). 18454356 10.1055/s-2008-1076753

[b15] Burger H. K. *et al.* Vascularized medial femoral trochlea osteochondral flap reconstruction of advanced Kienbock disease. J Hand Surg Am. 39, 1313–22 (2014). 24855965 10.1016/j.jhsa.2014.03.040

[b16] Yilmaz M. *et al.* Free arterialised venous forearm flaps for limb reconstruction. Br J Plast Surg. 49, 396–400 (1996). 8881787 10.1016/s0007-1226(96)90009-0

[b17] Koch H. *et al.* Clinical application of the retrograde arterialized venous flap. Microsurgery. 24, 118–24 (2004). 15038016 10.1002/micr.20011

[b18] Yan H. *et al.* Reconstruction of large dorsal digital defects with arterialized venous flaps: our experience and comprehensive review of literature. Ann Plast Surg. 70, 666–71 (2013). 23364671 10.1097/SAP.0b013e3182433575

[b19] Tan M. P., Lim A. Y. & Zhu Q. A novel rabbit model for the evaluation of retrograde flow venous flaps. Microsurgery. 29, 226–31 (2009). 19205061 10.1002/micr.20610

[b20] Liu Y. *et al.* A comparative study of four types of free flaps from the ipsilateral extremity for finger reconstruction. PLoS One. 9, e104014 (2014). 25098605 10.1371/journal.pone.0104014 PMC4123926

